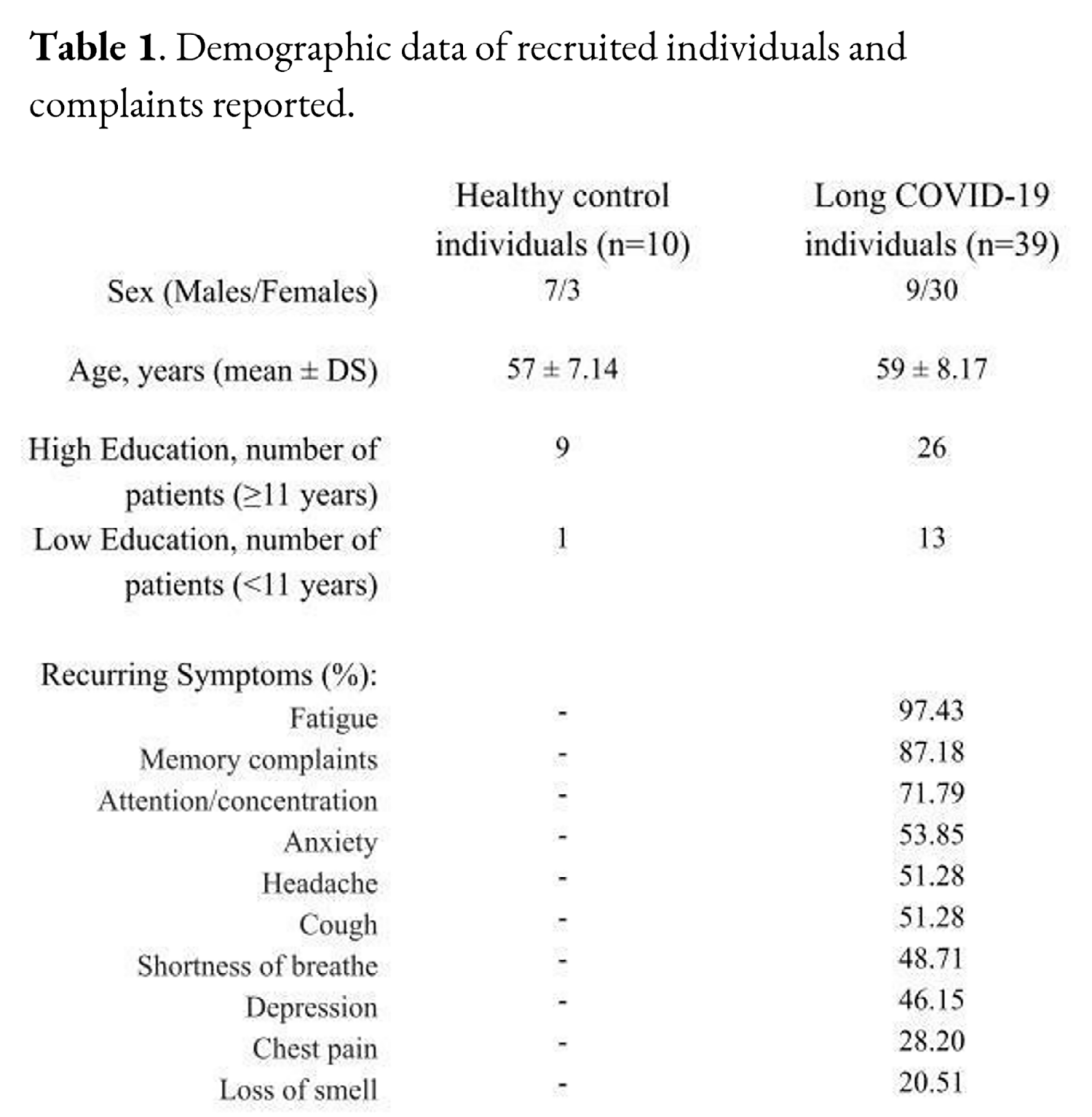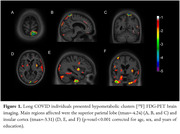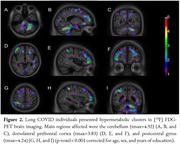# Brain Glucose Metabolism Changes in Long‐COVID: A Brazilian Cohort Study

**DOI:** 10.1002/alz.091969

**Published:** 2025-01-09

**Authors:** Maiele Dornelles Silveira, Luiza Santos Machado, João Pedro Ferrari‐Souza, Marco Antônio de Bastiani, Guilherme Povala, Wyllians Vendramini Borelli, Joana Emilia Senger, Ana Paula Bornes da Silva, Guilherme Bastos de Mello, João Pedro Uglione da Ros, Arthur Viana Jotz, Matheus Fakhri Kadan, Tharick A. Pascoal, Pedro Rosa‐Neto, Artur Francisco Schumacher‐Schuh, Cristina Sebastião Matushita, Graciane Radaelli, Mychael V Lourenco, Ricardo Benardi Soder, Artur Martins Coutinho, Daniele de Paula de Paula Faria, Jaderson Costa da Costa, Diogo O. Souza, Débora Guerini de Souza, Eduardo Rigon Zimmer

**Affiliations:** ^1^ Federal University of Rio Grande do Sul, Brazil, Porto Alegre, Rio Grande do Sul Brazil; ^2^ Federal University of Rio Grande do Sul, Porto Alegre, Rio Grande do Sul Brazil; ^3^ Universidade Federal do Rio Grande do Sul, Porto Alegre, Rio Grande do Sul Brazil; ^4^ University of Pittsburgh, Pittsburgh, PA USA; ^5^ Clinical Hospital of Porto Alegre, Porto Alegre, Rio Grande do Sul Brazil; ^6^ Federal University of Rio Grande do Sul, Brazil, Porto Alegre, RS Brazil; ^7^ Pontifícia Universidade Católica do Rio Grande do Sul, Porto Alegre, Rio Grande do Sul Brazil; ^8^ Lutheran University of Brazil, Canoas, Rio Grande do Sul Brazil; ^9^ Federal University of Rio Grande do Sul, Porto Alegre, RS Brazil; ^10^ Translational Neuroimaging Laboratory, The McGill University Research Centre for Studies in Aging, Montréal, QC Canada; ^11^ HCPA, Porto Alegre, Rio Grande do Sul Brazil; ^12^ Brain Institute of Rio Grande do Sul (BraIns), PUCRS, Porto Alegre, RS Brazil; ^13^ Brain Institute, RS, Porto Alegre, Rio Grande do Sul Brazil; ^14^ Institute of Medical Biochemistry Leopoldo de Meis, Federal University of Rio de Janeiro, Rio De Janeiro, Rio de Janeiro Brazil; ^15^ Pontifical Catholic University of Rio Grande do Sul, PORTO ALEGRE, RIO GRANDE DO SUL Brazil; ^16^ Center of Nuclear Medicine, Institute of Radiology, Hospital das Clínicas, Faculdade de Medicina da Universidade de São Paulo, São Paulo, São Paulo Brazil; ^17^ University of São Paulo Medical School, São Paulo, São Paulo Brazil; ^18^ Brain Institute of Rio Grande do Sul ‐ Pontifícia Universidade Católica do Rio Grande do Sul, Porto Alegre, Rio Grande do Sul Brazil; ^19^ UFRGS, Porto Alegre Brazil; ^20^ Federal University of Rio Grande do Sul (UFRGS), Porto Alegre, RS Brazil; ^21^ Brain Institute of Rio Grande Do Sul, PUCRS, Porto Alegre, RS Brazil

## Abstract

**Background:**

The COVID‐19 pandemic is a public health crisis, and its lasting consequences are not yet fully understood. Epidemiological data suggest that low‐ and middle‐income countries, such as Brazil, will bear a considerable burden of COVID‐19‐related comorbidities. Individuals who have survived COVID‐19 often report persistent symptoms, including neurological manifestations such as brain fog. However, the underlying brain biological changes behind the neurological symptoms remain undefined. Here, our goal was to assess the influence of long‐COVID on brain metabolism in adults from a Brazilian cohort.

**Method:**

Brazilian individuals (n=49) were recruited, examined through clinical and neuropsychological assessments, and divided into Control and Long‐COVID groups. Then, they underwent a brain [^18^F]FDG‐PET scan. Images were normalized by the global mean. Differences between groups were assessed through a [^18^F]FDG‐PET voxel‐wise linear regression accounting for age, sex and years of education (Table 1). The analysis was corrected for multiple comparisons using the cluster‐wise random field theory method (significant t < ‐3.28 and t > 3.28).

**Result:**

Long‐COVID group exhibited recurring symptoms such as fatigue, memory complaints and lack of concentration (Table 1). They also exhibited hypometabolic clusters in the left superior parietal lobe (t_max_=‐4.03; p < 0.001) and right insular cortex (t_max_=‐3.31; p < 0.001) (Figure 1). Hypermetabolic clusters were found in the left whole cerebellum (t_max_=4.92; p < 0.0001), right dorsolateral prefrontal cortex (t_max_=3.83; p < 0.001), and right postcentral gyrus (t_max_=4.24; p < 0.001) (Figure 2).

**Conclusion:**

Our preliminary results demonstrate region‐dependent dual response on brain metabolism due to Long‐Covid. More specifically, glucose hypometabolism was found in regions associated with cognitive domains and affective modulation. By contrast, glucose hypermetabolism was observed in brain areas associated with motor coordination and sensory processing. Metabolic changes in these regions should be further evaluated to advance in the understanding of the pathophysiological mechanisms associated with persistent neurological manifestations seen in individuals presenting with Long COVID.